# Hyperglycemia from Diabetes Potentiates Uncarboxylated Osteocalcin-Stimulated Insulin Secretion in Rat INS-1 Pancreatic β-Cells

**DOI:** 10.3390/nu16152384

**Published:** 2024-07-23

**Authors:** Pilailak Channuwong, Victoria Speight, Yuanying Yuan, Shaomian Yao, Masami Yoshimura, Fernando V. Bauermann, Ashish Ranjan, Sirichai Adisakwattana, Henrique Cheng

**Affiliations:** 1 Center of Excellence in Phytochemical and Functional Food for Clinical Nutrition, Department of Nutrition and Dietetics, Faculty of Allied Health Sciences, Chulalongkorn University, Bangkok 10330, Thailand; 2 Department of Physiological Sciences, College of Veterinary Medicine, Oklahoma State University, Stillwater, OK 74078, USA; 3 Department of Comparative Biomedical Sciences, School of Veterinary Medicine, Louisiana State University, Baton Rouge, LA 70803, USA; 4 Department of Veterinary Pathobiology, College of Veterinary Medicine, Oklahoma State University, Stillwater, OK 74078, USA; 5 Department of Radiation Oncology, UT Southwestern Medical Center, Dallas, TX 75390, USA

**Keywords:** uncarboxylated osteocalcin, pancreatic β-cells, hyperglycemia, calcium signaling, insulin secretion

## Abstract

Uncarboxylated osteocalcin (ucOC) is a hormone secreted by osteoblasts that strengthens bone during mineralization and is a biomarker for ongoing bone formation. It also regulates glucose homeostasis by stimulating insulin secretion from pancreatic β-cells. However, its effect on β-cells under hyperglycemic diabetic conditions is unclear. The objective of this study was to investigate ucOC’s effect on insulin secretion in β-cells maintained under high glucose conditions. We hypothesized that hyperglycemia potentiates insulin secretion in response to ucOC stimulation. Using INS-1 cells, we performed insulin secretion experiments, intracellular calcium recordings, and RT-qPCR to determine ucOC’s effect on glucose-stimulated insulin secretion (GSIS)-related genes. The results reveal that ucOC significantly increased insulin secretion under hyperglycemic conditions compared to lower glucose levels. High glucose conditions also potentiated the effect of ucOC on calcium signals, which enhanced insulin secretion. The increase in intracellular calcium was due to an influx from the extracellular space via voltage-dependent calcium channels (VDCCs). Interestingly, the treatment of cells with NPS-2143, a GPRC6A blocker, failed to abolish the calcium signals. Uncarboxylated osteocalcin upregulated the expression of GSIS-related genes under high glucose conditions (450 mg/dL) compared to cells under standard culture conditions (200 mg/dL). In conclusion, hyperglycemia potentiates ucOC-induced insulin secretion in β-cells by opening VDCCs and upregulating GSIS genes. These findings provide a better understanding of ucOC’s mechanism in the diabetic state and could lead to alternative treatments to stimulate insulin secretion.

## 1. Introduction

Diabetes mellitus is a major medical problem in need of new therapies. It afflicts 37.3 million Americans and 537 million people worldwide. Insulin secretion from pancreatic β-cells is essential for glucose homeostasis due to its ability to promote glucose uptake by cells and glycogen storage in the liver. These effects lower blood glucose and re-establish normoglycemia. Defects in insulin secretion can lead to diabetes mellitus, characterized by persistent hyperglycemia along with severe complications. Patients often develop kidney failure, heart disease, stroke, and even loss of limbs due to poor circulation. Furthermore, type 2 diabetic patients have lower ucOC levels compared to healthy individuals. While the importance of glucose-stimulated insulin secretion (GSIS) in blood sugar control is well known, various hormones contribute to insulin secretion [1]. This highlights the critical role of the endocrine system in regulating energy metabolism.

Osteocalcin is a peptide hormone secreted by osteoblasts into the blood in uncarboxylated form (ucOC). Despite its role in bone mineralization and being a marker of bone turnover, ucOC has emerged as a multifaceted hormone that exerts influence beyond the skeletal system [2]. Several studies have highlighted the importance of ucOC for glucose metabolism [3]. A crucial link between bone and pancreatic physiology revealed that ucOC stimulates insulin secretion from β-cells [4,5,6]. Clinical significance was established by the confirmation of lower ucOC blood levels in type 2 diabetes mellitus patients and proposed as a biomarker for glucose disturbances and cardiovascular risk in metabolic syndrome [7,8,9].

The mechanisms of ucOC in pancreatic islet cells during the diabetic state are not fully understood. Uncarboxylated osteocalcin is reported to stimulate insulin secretion by interacting with the G protein-coupled receptor family C group 6 member A (GPRC6A), a member of the G protein-coupled receptor family [10]. However, there are conflicting data showing an indirect effect of ucOC on β-cells by stimulating GLP-1 secretion from enterocytes, which in turn promotes insulin secretion [11]. In this study, ucOC failed to bind to and activate GPRC6A in β-cells. Others have identified GPR158 and GPR37 as ucOC receptors in brain neurons [12,13]. Experiments by Gao and colleagues provided insight between the interaction of glucose and ucOC in β-cells [14,15]. Uncarboxylated osteocalcin potentiates GSIS by closing ATP-sensitive potassium channels (K_ATP_), prolonging the action potential and increasing intracellular calcium [15]. The same group reported that the PLC and PKC signaling pathways mediate ucOC’s effects during GSIS [14]. Glucose alone is transported into β-cells by Glut2 transporters and metabolized by the enzyme glucokinase to generate energy in the form of ATP. The increase in ATP closes K_ATP_ channels, causing cell depolarization and opening of voltage-dependent calcium channels (VDCCs) and calcium influx [16,17]. The elevation in intracellular calcium triggers insulin secretion. In skeletal muscle, ucOC stimulates ATP production by enhancing mitochondrial activity [18]. Beyond its effect on ATP and calcium signaling, ucOC exerts a profound impact on gene expression profiles. Notably, ucOC enhances the expression of genes involved in glucose metabolism [5,19].

Understanding the interplay among ucOC, glucose, and insulin secretion holds significant clinical implications for metabolic disorders such as type 2 diabetes mellitus. Dysregulation of ucOC levels in diabetics highlights its potential as a therapeutic target for mitigating β-cell dysfunction and insulin resistance. We investigated the effect of ucOC on insulin secretion using INS-1 β-cells maintained under hyperglycemic conditions. We quantified insulin secretion in response to ucOC and performed intracellular calcium recordings to provide insight into its mechanism and RT-qPCR to determine its effect on genes controlling insulin secretion.

## 2. Materials and Methods

### 2.1. Reagents

All reagents were purchased from ThermoFisher Co. (Waltham, MA, USA), except that fura-2 acetoxymethyl ester (Fura-2AM) was from Cayman Chemical (Ann Arbor, MI, USA) and uncarboxylated [Gla17,21,24] osteocalcin (1–49) was purchased from AnaSpec Inc. (Fremont, CA, USA).

### 2.2. Cell Culture

Rat pancreatic β-cells (INS-1) were cultured in RPMI 1640 containing 200 mg/dL glucose (cat#22400-089) supplemented with 50 μM 2-mercaptoethanol, 1 mM sodium-pyruvate, 2 mM L-glutamine, and 10% fetal bovine serum (FBS). Glucose was added to glucose-free RPMI 1640 to obtain the desired concentration for experiments. Cells were maintained under 5% CO_2_ at 37 °C. Experiments were conducted using cells from passages 70–89.

### 2.3. Measurement of Insulin Secretion

Cells were plated into 24-well plates at ~10^5^ cells/well and grown for 4 days. Two days before the experiments (48 h), the glucose concentration was adjusted in individual wells between 0 and 450 mg/dL. Measurement of insulin secretion was accomplished by replacing the culture medium with modified Krebs–Ringer bicarbonate buffer (KRB) containing (in mM) NaCl 136, KCl 4.8, CaCl_2_ 2.5, KH_2_PO_4_ 1.2, MgSO_4_ 1.2, NaHCO_3_ 5, HEPES 10, 0.1% BSA, pH 7.4, and the respective glucose concentration. After a 30 min equilibration period at 37 °C, the KRB was removed and ucOC (100–1000 nM) prepared in KRB was added to stimulate insulin secretion. Cells were incubated with ucOC for an additional 30 min followed by KRB collection and storage at −80 °C for insulin quantification using an ultrasensitive rat insulin ELISA (Mercodia AB, Uppsala, Sweden). All experiments were performed in triplicates (3 wells/treatment group) and repeated with three different cell passages.

### 2.4. Real-Time Calcium Imaging Analysis

Intracellular calcium signals were obtained using a dual excitation fluorometric imaging system (Excelitas^®^ Technologies, Waltham, MA, USA) controlled by MetaFluor^®^ software version 7.10.5.487 (Molecular Devices, San Jose, CA, USA). Fura-2AM-loaded cells (2 μM/30 min/37 °C) were excited by wavelengths of 340 nm/380 nm and emission collected at 510 nm wavelengths. Fluorescence emissions of several cells were sampled and computed into relative ratio units of the fluorescence intensity of the different wavelengths (F340/F380). Data were presented as average calcium traces from several cells and peak increases showing the time point in which the highest calcium increase was recorded from each treatment group (mean and SEM).

### 2.5. RT-qPCR

Cells were maintained in 200 mg/dL glucose (culture conditions) or switched to 450 mg/dL glucose 2 days prior to ucOC treatment. Experiments were performed in 6-well plates (1 well/treatment group) and stimulated with ucOC for 4 days. The culture medium containing ucOC was replaced with fresh medium (450 mg/dL glucose) and hormone after 2 days of incubation. At the end of the fourth day, cells were collected with Trizol for total RNA extraction using bromochloropropane (BCP) separation and isopropanol precipitation method. The RNA was treated with Turbo^TM^ DNase digestion to eliminate possible DNA contamination and then assessed with a Nanodrop spectrometer to ensure OD260/280 > 1.8. Equal amounts of RNA from different treatments were reverse-transcribed into cDNA with M-MLV reverse transcriptase (Invitrogen, Waltham, MA, USA) following the manufacturer’s protocol. Next, the cDNA was mixed with an iTaq Universal SYBR Green Supermix (Bio-Rad, Hercules, CA, USA) and gene-specific rat primers for real-time PCR analysis to obtain CT values for calculation of relative gene expression using the ΔΔCT method normalized to β-actin. The experiments were repeated three times with different cell passages.
List of primers (forward/reverse [5′–3′]):CACCCAAGTCCCGTCGTGAAGT/GATCCACAATGCCACGCTTCTG (Ins)GAAATTCAAGAAGCGAAAAG/CCTGCTGTCACTCTGGTAGTAG (Cav_1.2_)TAAGGGGCACTGAGGACATC/TGCCAGCTGTCTGAAAAATG (GLUT2)AAGGGAACAACATCGTAGGA/CATTGGCGGTCTTCATAGTA (GK)TCCACCAGGTAGACATCCC/TAGGAGCCAGGTCGTAGAG (Kir_6.2_)GCGTGACATCAAAGAGAAG/ACTGTGTTGGCATAGAGG (β-actin)

### 2.6. Data Analysis

The effects of ucOC on insulin secretion under different glucose conditions and on relative gene expression were analyzed using unpaired Student’s *t*-test and peak calcium signals from imaging recordings with a one-way ANOVA followed by Tukey’s multiple comparisons test or Student’s *t*-test (GraphPad Prism version 10.0.3 (Boston, MA, USA)). Microsoft Excel^TM^ version 16.87 (Microsoft, Redmond, VA, USA) and SigmaPlot^®^ version 12.0 (Systat Software Inc., San Jose, CA, USA) were used for data organization and graph plotting. Statistical significance was established at *p* ≤ 0.05.

## 3. Results

### 3.1. High Glucose Concentrations Potentiated Osteocalcin-Induced Insulin Secretion

**Figure 1:**
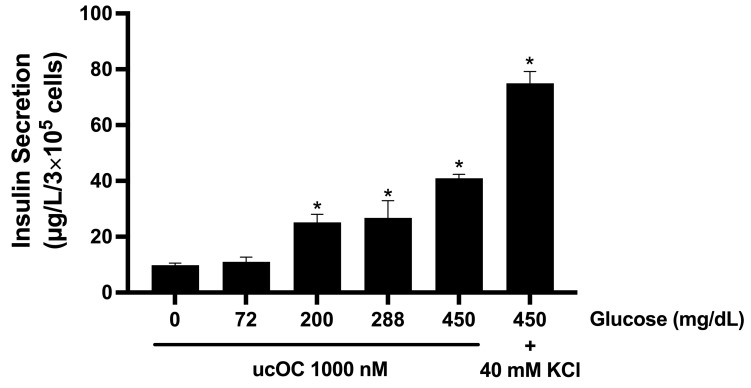
Effect of osteocalcin on insulin secretion under increasing glucose concentrations. Stimulation of INS-1 cells with ucOC increased insulin secretion in a glucose-dependent manner. Glucose potentiation of ucOC-stimulated insulin secretion could be noticed at 200 mg/dL glucose or higher. As a positive control, cells were depolarized with 40 mM KCl to stimulate insulin secretion. Results are expressed as mean + SEM; n = 3 wells/concentration from three independent experiments. * *p* ≤ 0.05 compared to normoglycemic conditions (72 mg/dL).

We first tested if increasing extracellular glucose concentrations in cultured cells would enhance the ability of ucOC to stimulate insulin secretion. The RPMI 1640 medium used for INS-1 cell culture contained 200 mg/dL glucose. Therefore, cells were incubated with glucose concentrations ranging from 0 to 450 mg/dL prior to hormone stimulation. Treatment of cells with 1000 nM ucOC increased insulin secretion in a glucose-dependent manner (Figure 1). At the highest glucose concentration (450 mg/dL), ucOC caused a 2-fold increase compared to culture conditions (200 mg/dL). No significant increases in insulin secretion were observed in cells stimulated with ucOC in the absence of glucose or under 72 mg/dL conditions (normoglycemia).

### 3.2. Osteocalcin Increased Insulin Secretion in a Concentration-Dependent Manner

**Figure 2:**
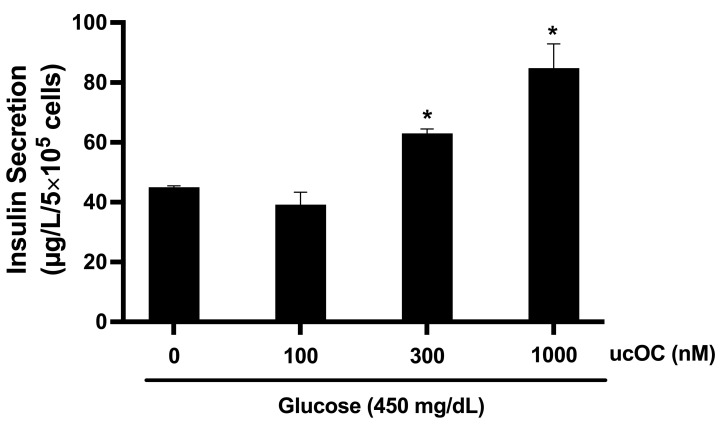
Dose–response for osteocalcin on insulin secretion under hyperglycemic conditions. Stimulation of INS-1 cells with increasing ucOC concentrations under high glucose conditions (450 mg/dL) increased insulin secretion in a concentration-dependent manner. Results are expressed as mean + SEM; n = 3 wells/concentration from three independent experiments. * *p* ≤ 0.05 compared to control cells in the absence of ucOC.

Based on our findings that hyperglycemia potentiated ucOC-induced insulin secretion, we performed experiments with increasing ucOC concentrations (100–1000 nM) under 450 mg/dL glucose conditions. Treatment of cells with ucOC stimulated insulin secretion in a concentration-dependent manner (Figure 2). At 300 nM and 1000 nM, ucOC increased insulin secretion 1.5- and 2-fold, respectively.

### 3.3. High Glucose Concentration Potentiated Osteocalcin-Induced Intracellular Calcium Signals

**Figure 3:**
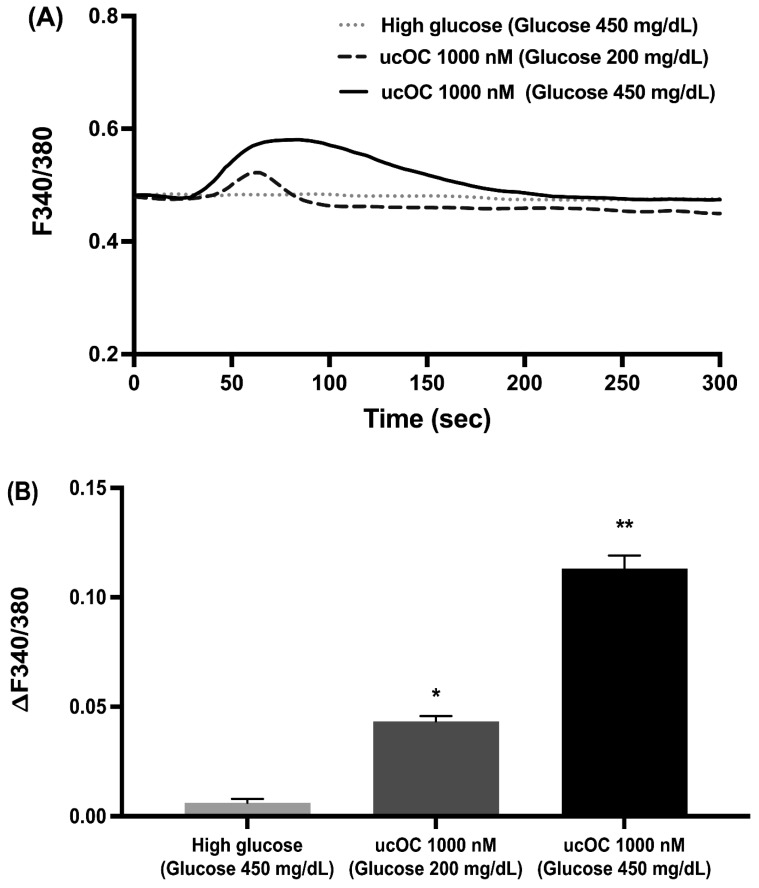
High glucose conditions potentiated osteocalcin-induced intracellular calcium signals. (**A**) Average intracellular calcium recordings from cells maintained in high glucose conditions (450 mg/dL) and stimulated with ucOC (n = 150 cells), from cells maintained in 450 mg/dL glucose alone (n = 50 cells), and from cells maintained in standard culture conditions (200 mg/dL) and stimulated with ucOC (n = 81 cells). (**B**) Average peak calcium responses from cells in panel A. Results are presented as average traces or peak responses (mean + SEM). * *p* ≤ 0.05 compared to 450 mg/dL glucose-alone group, ** *p* ≤ 0.05 compared to 200 mg/dL glucose group.

Elevations in intracellular calcium are a requirement for insulin secretion. Therefore, we examined if high glucose conditions (450 mg/dL) potentiated the calcium signals generated by ucOC. Stimulation of cells with 1000 nM ucOC after 2 days in 450 mg/dL glucose increased intracellular calcium compared to cells in 200 mg/dL glucose or stimulation with 450 mg/dL glucose alone (Figure 3A). The peak calcium increase for each treatment group is shown in Figure 3B. In the experiment, cells that maintained under 450 mg/dL glucose had greater calcium responses to ucOC compared to those in 200 mg/dL.

### 3.4. Extracellular Calcium Is Required for the Osteocalcin Response

**Figure 4:**
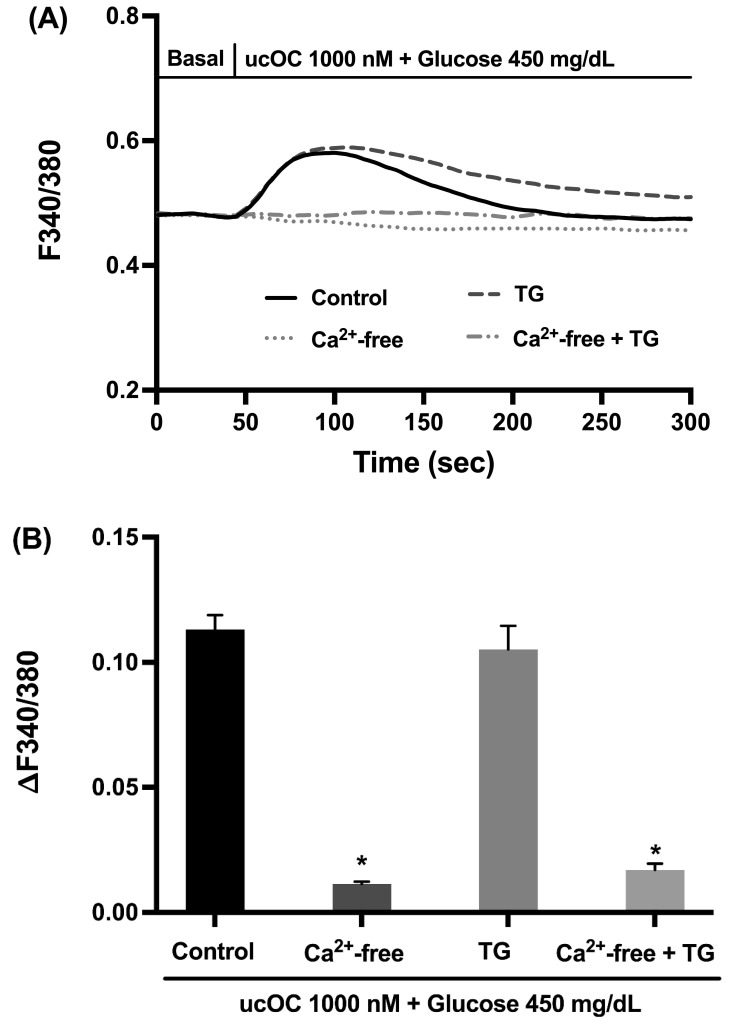
Calcium source for osteocalcin-increased intracellular signals. (**A**) Average intracellular calcium recordings from cells treated with thapsigargin (TG) (n = 52 cells) and/or maintained in extracellular calcium-free buffer (n = 150 and 120 cells, respectively). In the absence of extracellular calcium, ucOC failed to generate calcium signals, but not after endoplasmic reticulum depletion. (**B**) Average peak calcium responses from cells in panel A. Note that absence of extracellular calcium abolished the ucOC response, with or without thapsigargin. Results are presented as average traces or peak responses (mean + SEM). * *p* ≤ 0.05 compared to the control group (n = 150 cells) in the presence of extracellular calcium and without thapsigargin treatment.

Intracellular calcium signals can originate from internal stores (e.g., endoplasmic reticulum) and/or influx from the extracellular space. Hence, we performed experiments to determine the calcium source for the ucOC responses using thapsigargin to deplete the endoplasmic reticulum and extracellular calcium-free conditions. Treatment of cells with thapsigargin had no effect on the calcium signals by ucOC, while the absence of extracellular calcium or in combination with thapsigargin abolished the calcium signals (Figure 4A). The peak calcium increases for each treatment group demonstrated the importance of calcium influx for the effect of ucOC in β-cells (Figure 4B).

### 3.5. Voltage-Dependent Calcium Channels Are Activated in Response to Osteocalcin

**Figure 5:**
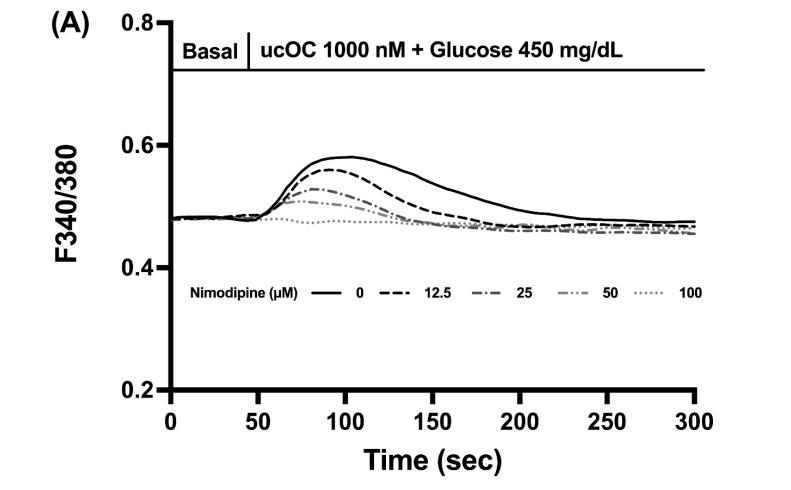

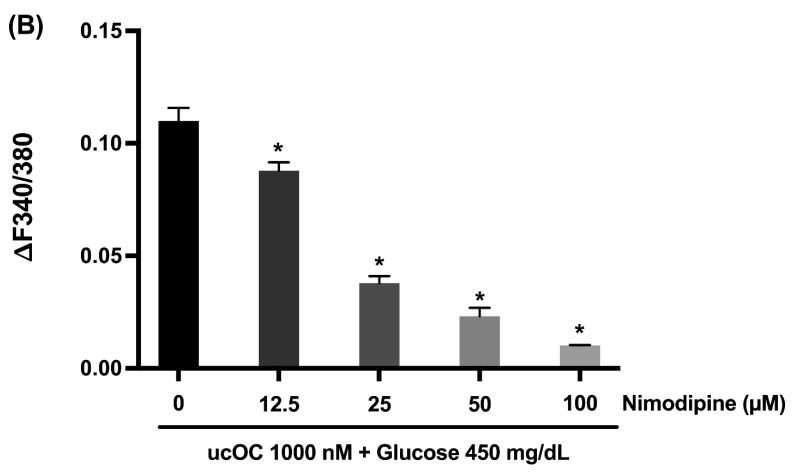
Osteocalcin activated voltage-dependent calcium channels in β-cells. (**A**) Average intracellular calcium recordings from cells treated with increasing nimodipine concentrations (0 µM n = 150 cells; 12.5 µM n = 76 cells; 25 µM n = 130 cells; and 100 µM n = 152 cells) and stimulated with ucOC. (**B**) Average peak calcium responses from cells in panel A. Nimodipine inhibited the calcium signals by ucOC in a concentration-dependent manner. Results are presented as average traces or peak responses (mean + SEM). * *p* ≤ 0.05 compared to the control group without nimodipine treatment.

Calcium influx into β-cells is mainly due to the opening of L-type VDCCs. Utilizing nimodipine, a VDCC blocker, we tested if these channels were responsible for the elevations in intracellular calcium by ucOC. Treatment of cells with nimodipine (12.5–100 µM) inhibited the calcium signals in a concentration-dependent manner (Figure 5A). The reduction in peak calcium signals with increased nimodipine concentration can be seen in Figure 5B.

### 3.6. Osteocalcin Receptor in Pancreatic β-Cells

**Figure 6:**
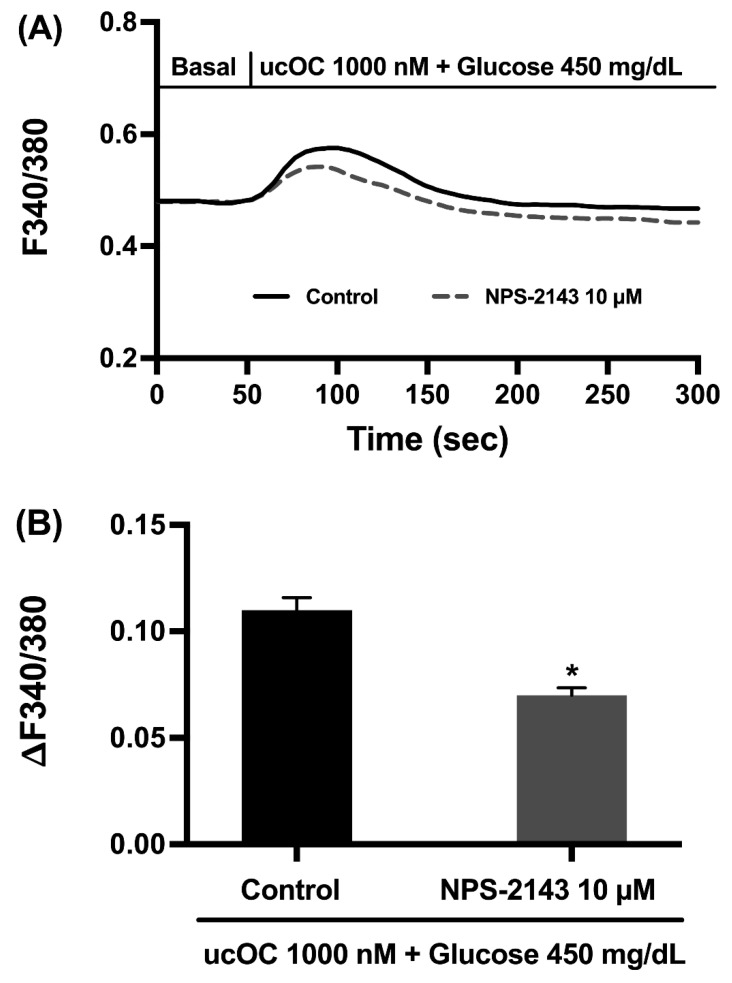
Involvement of GPRC6A on osteocalcin-increased intracellular calcium signals. (**A**) Average intracellular calcium recordings from cells treated with NPS-2143, an antagonist for the GPRC6A receptor, followed by ucOC stimulation under high glucose conditions (n = 136 cells). (**B**) Average peak calcium responses from cells in panel A. Results are presented as average traces or peak responses (mean + SEM). * *p* ≤ 0.05 compared to the control group without NPS-2143 treatment (n = 150 cells).

The effects of ucOC on β-cells are suggested to be mediated by GPRC6A, a member of the G protein-coupled receptor family [10,20]. Therefore, we tested if NPS-2143, a non-competitive antagonist of GPRC6A could inhibit the calcium signals by ucOC under high glucose conditions. Studies show that at a 10 µM concentration, NPS-2143 blocks the effects of ucOC via GPRC6A [21,22]. In our experiments, INS-1 cells pretreated with 10 µM NPS-2143 for 20 min remain capable of responding to an ucOC stimulus with a 64% increase in the peak calcium signal (Figure 6A,B).

### 3.7. Osteocalcin Upregulated Glucose-Stimulated Insulin Secretion-Related Genes

**Figure 7:**
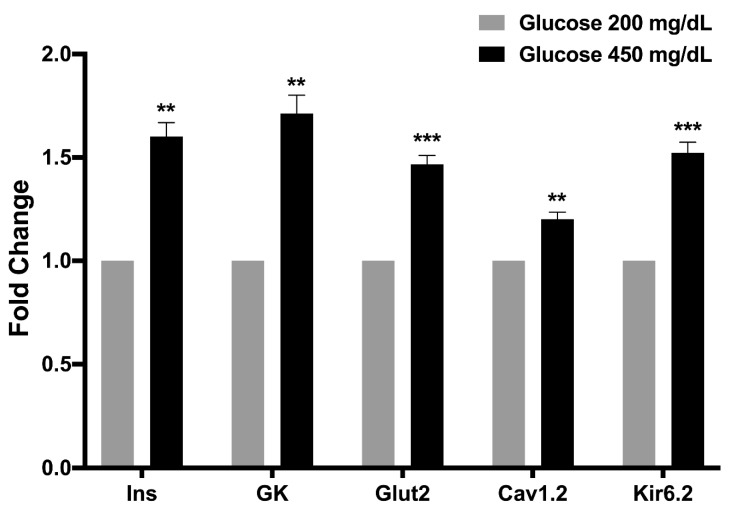
Effect of osteocalcin on glucose-stimulated insulin secretion-related genes. Uncarboxylated osteocalcin upregulated the expression of glucose-stimulated insulin secretion-related genes in cells maintained under hyperglycemic conditions (450 mg/dL) compared to cells in culture conditions (200 mg/dL glucose). Cells were treated with ucOC for four days before RT-qPCR analysis. Results are expressed as mean + SEM from three different cell passages (n = 3). Data were normalized with β-actin as an internal control. ** *p* ≤ 0.01 and *** *p* ≤ 0.001 compared to 200 mg/dL glucose.

To determine whether ucOC controlled the expression of key insulin secretion genes, INS-1 cells maintained under hyperglycemic conditions (450 mg/dL) were stimulated during four consecutive days. At the end, the expression of the insulin (Ins), glucose transporter 2 (Glut2), glucokinase (GK), VDCC (Cav_1.2_), and K_ATP_ (Kir_6.2_) genes were quantified and compared to cells cultured under standard glucose conditions (200 mg/dL). In the presence of high glucose (450 mg/dL), ucOC increased the expression of all genes examined compared to cells in 200 mg/dL glucose (Figure 7). In the experiment, no significant differences in gene expression were observed between the two glucose concentrations without ucOC in the culture medium. 

## 4. Discussion

We examined the effect of diabetic hyperglycemia on ucOC-stimulated insulin secretion in INS-1 β-cells. Cells were treated with ucOC after being maintained in high glucose conditions. The results reveal that persistent high glucose conditions potentiated the effects of ucOC on insulin secretion, and this effect was dose-dependent. To obtain insight into ucOC’s mechanism of action, we performed intracellular calcium recordings and a gene expression analysis. In addition to insulin secretion, hyperglycemia potentiated the increases in intracellular calcium signals by ucOC compared to cells maintained at lower glucose levels. These results demonstrate that the increase in insulin secretion was caused by further elevations in intracellular calcium. Interestingly, ucOC potentiates GSIS when applied simultaneously to β-cells [15]. This mechanism involves closure of the K_ATP_ channel, action potential prolongation, and cell depolarization. In this scenario, ucOC functions to amplify the glucose stimulus that relies on glucose uptake into β-cells to generate ATP, resulting in K_ATP_ closure and cell depolarization [16]. The depolarization activates VDCCs, leading to a calcium influx, and ultimately insulin secretion [17]. Based on our findings and those reported by Gao and colleagues, it appears that calcium influx into β-cells is a requirement for insulin secretion by ucOC but not for release from internal stores. Furthermore, our studies show that β-cells maintained under hyperglycemic conditions become more responsive to ucOC, suggesting that GSIS and a persistent high glucose environment potentiate ucOC’s effect on calcium signaling and insulin secretion.

Mechanistic studies for ucOC in β-cells revealed the involvement of the PLC-PKC pathway [14]. The activation of PLC also generates inositol triphosphate (IP_3_), which increases intracellular calcium by promoting its release from the endoplasmic reticulum. Our data show that the depletion of endoplasmic reticulum calcium with thapsigargin failed to impact calcium signaling by ucOC, which ruled out the involvement of the PLC-IP_3_ pathway in its mechanism. Although controversial, studies indicate that ucOC activates GPRC6A in β-cells [10,20]. We examined if this type of G protein-coupled-receptor mediated ucOC’s effect under high glucose conditions with the GPRC6A antagonist, NPS-2143 [21,22]. Treatment of cells with NPS-2143 partially blocked the calcium signals. These results suggest that neither the PLC-IP_3_ pathway nor GPRC6A receptors are directly involved in the mechanism of ucOC in β-cells during hyperglycemia. This agrees with a study showing that ucOC does not interact with GPRC6A in β-cells [11]. Others reported an indirect effect of ucOC by stimulating GLP-1 secretion from enterocytes, which bind to its receptor in β-cells to promote insulin secretion [23]. In our case, it is unlikely to be an indirect effect of ucOC, since INS-1 cells were used for experiments. Nonetheless, it is possible that ucOC binds to a different receptor type. Two isoforms for the ucOC receptor, GPR158 and GPR37, have been reported in brain neurons [12,13].

Since INS-1 cells maintained under high glucose conditions became more responsive to ucOC and due to its ability to potentiate GSIS [15], we performed a gene expression analysis to determine whether ucOC controlled the expression of genes involved in insulin secretion. The stimulation of cells maintained in hyperglycemic conditions significantly upregulated the expression of GSIS-related genes, especially those involved in controlling cell depolarization and calcium influx (Kv_1.2_ and Cav_1.6_ channels). In the absence of ucOC, high glucose levels of 450 mg/dL did not affect GSIS-related gene expression compared to cells maintained at 200 mg/dL glucose. Because one of the functions of ucOC is to stimulate insulin secretion, it is reasonable to speculate that under hyperglycemic conditions, such as diabetes mellitus, the upregulation of GSIS-related genes and perhaps others would be an attempt by β-cells to generate mechanisms to lower blood glucose levels. This is supported by findings in β-cells and adipocytes where ucOC upregulates the expression of genes involved in glucose metabolism, including those for glucose transport and insulin itself [5,19].

## 5. Conclusions

In conclusion, the results demonstrate in INS-1 β-cells that hyperglycemic conditions, like those present in diabetes mellitus, potentiated the effects of ucOC on intracellular calcium signaling and insulin secretion. Under high glucose conditions, several genes involved in insulin secretion were upregulated by ucOC. From our observations and those of others, it appears that ucOC’s mechanism involves mainly calcium influx rather than its release from intracellular stores. Understanding the intricate interplay between ucOC signaling and calcium dynamics in β-cells provides valuable insights into the regulation of glucose homeostasis and the pathophysiology of metabolic disorders such as type 2 diabetes mellitus. Further studies in pancreatic islets and diabetic animal models are required to confirm our findings in INS-1 cells. Moreover, exploring the intricate mechanisms underlying ucOC’s effects on insulin secretion may unveil novel therapeutic approaches using the hormone to enhance β-cell function and preserve metabolic health.

## Data Availability

The original contributions presented in the study are included in the article, further inquiries can be directed to the corresponding author.

## Abbreviations

ucOC	Uncarboxylated osteocalcin
GSIS	Glucose-stimulated insulin secretion
VDCC	Voltage-dependent calcium channel
PLC	Phospholipase C
PKC	Protein kinase C
KRB	Krebs–Ringer bicarbonate buffer
GK	Glucokinase
Cav_1.2_	L-type Ca^2+^ channel
Ins	Insulin
GLUT2	Glucose transporter 2
K_ATP_	ATP-sensitive K^+^ channel
IP_3_	Inositol triphosphate
GPRC6A	G protein-coupled receptor family C group 6 member A
